# Conformational Flexibility of the Protein Insertase BamA in the Native Asymmetric Bilayer Elucidated by ESR Spectroscopy

**DOI:** 10.1002/anie.202113448

**Published:** 2021-12-02

**Authors:** Aathira Gopinath, Benesh Joseph

**Affiliations:** ^1^ Institute of Biophysics Department of Physics Center for Biomolecular Magnetic Resonance (BMRZ) Goethe University Frankfurt Max-von-Laue-Str. 1 60438 Frankfurt/Main Germany

**Keywords:** conformational dynamics, membrane proteins, PELDOR/DEER spectroscopy, protein structures, structural biology

## Abstract

The β‐barrel assembly machinery (BAM) consisting of the central β‐barrel BamA and four other lipoproteins mediates the folding of the majority of the outer membrane proteins. BamA is placed in an asymmetric bilayer and its lateral gate is suggested to be the functional hotspot. Here we used in situ pulsed electron‐electron double resonance spectroscopy to characterize BamA in the native outer membrane. In the detergent micelles, the data is consistent with mainly an inward‐open conformation of BamA. The native membrane considerably enhanced the conformational heterogeneity. The lateral gate and the extracellular loop 3 exist in an equilibrium between different conformations. The outer membrane provides a favorable environment for occupying multiple conformational states independent of the lipoproteins. Our results reveal a highly dynamic behavior of the lateral gate and other key structural elements and provide direct evidence for the conformational modulation of a membrane protein in situ.

## Graphical abstract

The outer membrane of Gram‐negative bacteria is an asymmetric bilayer. The effect of this asymmetry on protein structure and dynamics remains elusive. The β‐barrel assembly machinery (BAM) mediates the folding and insertion of proteins into the outer membrane. Using in situ electron spin resonance spectroscopy, we show that the native outer membrane significantly enhances the dynamics of the BamA insertase of the BAM complex.

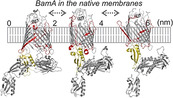

## Introduction

**Figure 1:**
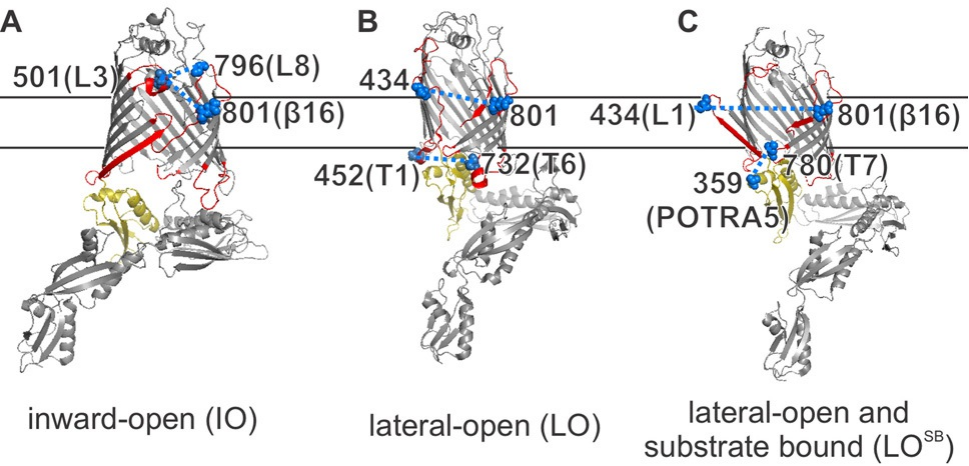
Lateral gate conformations of BamA. The key structural elements investigated here are highlighted in red (L1, L3, L8, β16, T1, and T6) or light yellow (POTRA5). A) In the inward‐open state (IO, PDB 5D0O), the β1 and β16 strands are zipped, leaving the barrel open to the periplasm. B) In the lateral‐open state (LO, PDB 5LJO), the β1 (to β6), L1, and the L3 move apart and T1, T6, and POTRA5 adopt a closed conformation with respect to the barrel. C) In the lateral‐open substrate bound state (LO^SB^, PDB 6V05), β1–β16 separation further increases, and L3 moves back into the barrel lumen. The lipoproteins or the substrate are not shown for clarity. Spin‐labeled positions are highlighted as spheres (in blue).

The cell envelope of Gram‐negative bacteria comprises of an inner membrane and an outer membrane (OM) separated by the periplasm. The OM is an asymmetric bilayer with a phospholipid‐ and a lipopolysaccharide (LPS) leaflet and carries numerous β‐barrel proteins, which are essential for survival. Additionally, LPS carries the O‐antigen, which consists of repeating oligosaccharide subunits. Overall, the effect of such membrane asymmetry on protein structure and dynamics remains elusive. The BAM complex, which is located in the OM (MW of ≈200 kDa) mediates the folding and insertion of most of the outer membrane proteins (OMPs).^[1]^ The central β‐barrel BamA contains five polypeptide transport‐associated domains (POTRA 1–5), which interact with the four lipoproteins (BamB‐E) to form a ring‐like structure in the periplasm. BamA and BamD are essential and conserved. BamB, C, and E are not present in all species and are suggested to perform an accessory role in the assembly pathway. Despite the enormous diversity of OMPs in *E. coli*, only BamA and the lipopolysaccharide insertase LptD are essential, and they attracted huge interest as the target for novel drugs. Available structural and biophysical data show that BamA barrel can exist in two major conformations: an inward‐open (IO or laterally‐closed) and a lateral‐open (LO) state (Figure 1 A–C).[^2^, ^3^, ^4^, ^5^, ^6^, ^7^] In the IO state, the last β16 strand exists in a fully zipped conformation, whereas in the LO state, it is in a kinked state. The lateral opening further increases when the substrate is bound (denoted as LO^SB^).^[8]^

For the OM, the outer LPS layer might be less fluid as compared to the inner phospholipid leaflet.^[9]^ The OM is a protein rich bilayer with a low lipid/protein ratio. The OMPs interact with each other to form clusters and organize into “OMP islands” with low mobility.^[10]^ Overall, this creates an unfavorable environment and a significant energy barrier for the insertion of newly forming OMPs. BAM is suggested to lower this energy barrier through a “membrane disruptase” or “lipid disorderase” activity.[^11^, ^12^, ^13^] However, the inability to reconstitute BAM into the native asymmetric OM hindered an experimental validation of this hypothesis and a thorough understanding of BAM function.

Electron spin resonance (ESR) spectroscopy, in particular, pulsed electron‐electron double resonance (PELDOR or DEER) spectroscopy has emerged as a powerful tool to study protein complexes, even in the cellular environments.[^14^, ^15^, ^16^, ^17^, ^18^, ^19^, ^20^, ^21^] PELDOR data reveals the ensemble conformational heterogeneity and in favorable cases can resolve the thermodynamic and kinetic aspects with spatiotemporal resolution.[^22^, ^23^, ^24^, ^25^, ^26^, ^27^] The nitroxide‐based methane thiosulfonate spin label (MTSL) is the most preferred tag for proteins.[^28^, ^29^] Other spin labels such as shielded nitroxides, Cu^II^, Gd^III^, and trityl are getting very attractive, especially for *in cell* studies.[^15^, ^27^, ^29^, ^30^, ^31^, ^32^, ^33^, ^34^, ^35^]

## Results and Discussion

To observe the conformational heterogeneity in BamA, we engineered pairs of cysteine substitutions around the lateral gate (Figure 1 A–C). At the extracellular side, β16 strand (at position Q801C) was paired with loop 1 (L1, at position T434C) or loop 3 (L3 at position L501C). The L3 was additionally paired with loop 8 (L8, at position G796C). At the periplasmic side, turn 1 (T1, at position T452C) was related to turn 6 (T6, at position S732C). To monitor orientation of the POTRA5, position T359C was paired with turn 7 (T7, at position L780C). In the lauryldimethylamine‐*N*‐oxide (LDAO) detergent micelles, all the BamA variants could be labeled using MTSL (forming the side chain denoted as R1) with high efficiency (≥70 %). PELDOR samples were prepared directly from the size‐exclusion chromatography (SEC) fractions at the low micromolar concentration (20–40 μM, Figures S1 and S2). Isolation of the native OM and spin labeling were performed following the protocols we previously established (Figures S2 and S3).^[22]^ The SDS‐PAGE and Western blot analysis confirmed the presence of BamA in the native OM (Figure S3). Colony growth assays revealed that the cysteine substitutions do not affect the function (Figure S4). The modulation depth (*Δ*) for the PELDOR data (10–20 %, in Figure 3, with a *Δ*_max_≈30 % under our experimental set up) revealed an effective labeling efficiency in the range of 30–70 % in the native OM.

In the LDAO micelles, spin labeled BamA gave a phase memory time (*T*_M_) of ≈3.0 μs (Figure S5). In the OM, MTSL labels gave a *T*_M_ of ≈1–1.5 μs, which is typical for membrane reconstituted samples. The spin labeled native OM sample contains some background signals (Cys‐less in Figure S2), which do not interfere with the distance measurements, but reduces the overall sensitivity to some extent by lowering the effective modulation depth.^[22]^ OMPs rarely have reactive cysteines and small background labeling could occur through physical adsorption of the MTSL labels with the membranes. Earlier, for the cobalamin transporter BtuB, we showed that the OM preparations of single cysteine variants do not give any distances, whereas clear dipolar modulation can be observed between spin pairs engineered within BtuB.[^19^, ^20^, ^22^] For BamA as well, the singly labeled variants gave a characteristic stretched exponential decay devoid of any distances (Figure S6A). Also, there is no significant instantaneous diffusion of the spins in the OM samples (Figure S5, bottom rightmost panel). Thus, overexpression does not cause oligomerization or aggregation of BamA in the native membranes.

**Figure 2:**
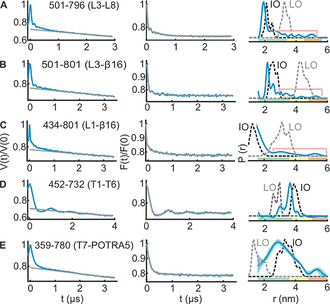
PELDOR spectroscopy of BamA in LDAO micelles. A–E) Left panels: primary PELDOR data (blue) overlaid with the intermolecular (background) contribution (in grey); middle panels: the background‐corrected form factors with the fit (in blue and grey, respectively); right panels: the determined distance distributions using Tikhonov regularization. Distances marked with pink lines suggest the presence of longer distances (see Figures S7 and S8). The error bars show the full variation of the probability for the given distances corresponding to the uncertainty in the background function (see Table S1). Additionally, the color code relates the reliability for different features of the probability distribution with the length of the observed dipolar evolution time. In the green zone, shape, width, and the mean distance are accurate. In the yellow zone, width and the mean, and in the orange zone, the mean distance are reliable. Simulations on the IO and LO structures are overlaid (dotted lines).

**Figure 3:**
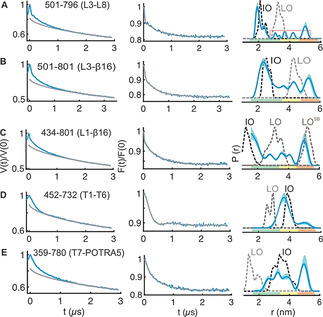
PELDOR spectroscopy of BamA in the native outer membranes. A–E) Left panels: primary PELDOR data (blue) with the intermolecular contribution (in grey); middle panels: the background‐corrected form factors with the fit (in blue and grey, respectively); right panels: the determined distance distributions using Tikhonov regularization. Distances indicated with the pink line (in A) are additionally narrowed during TR (Figure S9A). The error bars show the full variation of probability (see Table S1). Color codes for the probability distribution are as explained in Figure 2. Simulations on the IO, LO, or the LO^SB^ structures are overlaid (dotted lines).

To compare the experimental data with the available structures, we simulated the corresponding interspin distances on the IO and the LO structures using a rotamer library for MTSL labeled cysteines in proteins (overlaid in Figure 2 A–E).^[36]^ Simulations show that the engineered spin pairs can clearly resolve between these conformations. For the lateral gate, the transition from the LO to the LO^SB^ states is further resolved from the L1‐β16 (434–801) distances (Figure 3 C). We experimentally determined the dipolar coupling for all the spin pairs in detergent micelles and the native OM using PELDOR spectroscopy and the interspin distances were calculated employing Tikhonov regularization (TR) and deep neural network processing.[^37^, ^38^]

Our results from two independent sets of experiments show that in LDAO micelles BamA barrel predominantly exists in a conformation very similar to the IO state (Figure 2; Figures S7 and S8). At the extracellular side, L3‐L8, L3‐β16, and L1‐β16 data (Figure 2 A–C) are in quite good agreement with the simulations on the IO state. Yet, there are small peaks with lower amplitudes at longer distances (indicated with pink lines). For L3‐L8 and L3‐β16, those peaks might be narrowed due to the small regularization parameter (α) required to fit the major (and the narrow) distance peak. This is expected for TR when the data contains distance peaks with different widths. Further simulations as well as analysis with a higher α suggested a broad distribution in this range (Figure S7A–F and Figure S8A–D). This is also evident in the results obtained with the deep neural network processing (Figure S7K). Thus, L3 predominantly adopts the closed (IO) conformation (Figure 1), and the small fraction of broad distances might arise from the dynamics of these structural elements. The L1‐β16 data as well revealed a similar behavior with a major peak close to the IO conformation and additional small peaks corresponding to the LO state (Figure 2 C, Figure S7K). Considering the rather low amplitude of the distances (and the limited reliability for the exact distance, which are indicated with the pink lines), we interpret them merely as a qualitative reflection of the underlying dynamics of these structural elements in a fraction of the BamA molecules. At the periplasmic side, the T1‐T6 distances (Figure 2 D) showed a *r*_max_ close to the simulation for the IO state. The T7‐POTRA5 data revealed a somewhat broader distribution (Figure 2 E), which might be due to the flexibility of the POTRA5 and or the T7. Nonetheless, the overall distribution is closer to the IO state.

The native OM significantly enhanced the conformational heterogeneity of BamA (Figure 3; Figures S9 and S10). The L3‐L8 data gave a distribution comparable with that observed in the LDAO micelles (Figure 3 A). As explained above for the LDAO data, the longer distance peaks are more narrowed during analysis using TR (Figure S9B,C). The broad nature of the distribution might reflect the flexibility for both L3 and L8 (Figure 1 and Figure S9C). Such an enhanced conformational heterogeneity is observed also from the L3‐β16 and L1‐β16 data (Figure 3 B,C). The results from deep neural network processing predicted a rather similar distance distribution and it accurately fitted the background functions, which we experimentally determined in the OM (Figure S9L). The L3‐β16 showed a major peak, which agrees with the simulation on the IO structure, but broader than the LDAO data. The additional distances gave a broad distribution surrounding the simulation for the LO structure. The position 801 shows minimal changes for its orientation between different conformations (Figure 1) and as it is located on the β16, it would have less flexibility (unlike position 796 on L8 for e.g.). Therefore, the L3‐β16 distances suggest a dynamic ensemble of L3 existing in a continuum of states covering the broad conformational space observed in the structures (Figure 1). The L1‐β16 data also revealed a very broad distribution (Figure 3 C; Figures S9F and S10D). The β16 and β1(to β6) move apart during transition from the IO to the LO state (Figure 1). Simulations gave distinct distances in the IO, LO, and the LO^SB^ states between L1 and β16 (Figure 3 C). Interestingly, the experimental distance distribution spans a broad range encompassing the peaks corresponding to all three states. Overall, the heterogeneity is significantly enhanced for the lateral gate and the L3 in the native membranes as compared with the LDAO micelles (Figure 3 A–C).

At the periplasmic side, the T1‐T6 data shows a major peak closer to the IO state (Figure 3 D) and broader than the distribution in LDAO micelles. The minor peak at ≈5 nm could be accommodated with a different side chain packing (Figure S9 H, rightmost panel). For the T7‐POTRA5 distances (Figure 3 E), the first peak agrees with the IO state and the second peak corresponds to an even longer distance between T7 and POTRA5. These latter distances cannot be fully accommodated with a rearrangement of the side chains (Figure S9K rightmost panel and Figure S9L bottom panel). As T7 is a very short turn and the POTRA5 might have only limited internal dynamics,^[39]^ this peak might arise from the flexibility of the POTRA5.^[40]^ Previous molecular dynamics (MD) simulations suggested that the POTRA domains have a higher flexibility in the absence of lipoproteins and sample more conformations than observed in the structures.[^2^, ^39^] In conclusion, our data validates the orientation of the POTRA5 corresponding to the IO structure and suggest additional flexibility in the native outer membranes.

**Figure 4:**
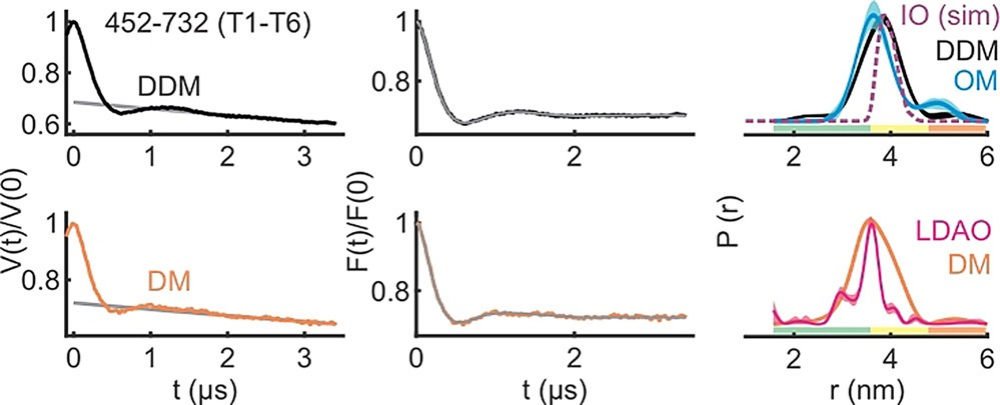
The inter‐turn (T1–T6) distances of BamA in DM, DDM, LDAO detergent micelles, and the native OM. Primary PELDOR data (left) with the intermolecular contribution (in grey), the background corrected form factors with the fit (middle), and the determined distance distributions using TR are shown (right). The LDAO distances are taken from Figure 2 D. The OM distances correspond to data presented in Figure 3 D. The error bars show the full variation of probability, which is invisible if smaller than the linewidth (see Supplementary Table S1). Color codes for the probability distribution are as explained in Figure 2. Simulation (sim) on the IO structure is overlaid (dotted line).

To further elucidate the effect of the surrounding environment, additional experiments were performed with the T1‐T6 pair in n‐decyl‐β‐D‐maltopyranoside (DM) and n‐Dodecyl‐β‐D‐maltopyranoside (DDM) detergent micelles. The distribution is the narrowest in the crystal structure followed with LDAO micelles (Figure 4). Interestingly, this structure has been solved in the mixed micelles of N‐nonyl‐β‐d‐glucoside (β‐NG) and tetraethylene glycol monooctyl ether (C_8_E_4_). The distance distribution gets broader in DM, DDM (with a small increase of the *r*_max_), and the native OM. Overall, the observed differences could be accommodated with a differential side chain packing (Figure S7J), suggesting that the different environments might modify the local surroundings of the observed positions on T1 and or T6. In line with a recent cryo‐EM investigation,^[41]^ our observations altogether show that the surrounding detergents and lipids can differentially modulate BamA dynamics both locally and globally (Figures 2–4).

Though OM model systems are being developed,^[42]^ experimental reconstitution of BamA/BAM into an asymmetric membrane is yet to be reported. The nature of the lipids,[^13^, ^43^] hydrophobic thickness,^[44]^ or membrane defects^[12]^ were shown to have profound effect on the folding activity of BamA. MD simulations of BamA from *Neisseria gonorrheae* and *Haemophilus ducreyi* in symmetric dimyristoyl‐phosphatidylethanolamine (DMPE) membranes showed lateral opening between β1 and β16.^[45]^ However, similar investigation of the full‐length BamA from *E. coli* in an asymmetric bilayer like the native OM did not show any such opening.^[39]^ In line with our spectroscopic observations (Figure 2), structures of BamA in LDAO^[46]^ micelles (also in C_8_E_4_ micelles,^[47]^ and DMPC:CHAPSO bicelles^[45]^) showed an inward‐open state of the barrel, suggesting it as the most stable state in these environments. Interestingly, cryo‐EM studies of BAM in nanodiscs revealed the BamA barrel exclusively in the lateral‐open conformation.[^13^, ^41^] Thus, the dynamics of BamA is highly influenced by the environment. Our results suggest that the native outer membrane creates a unique environment with a low energy barrier between different conformations to enable a highly dynamic behavior of the BamA barrel.

Under laboratory conditions, BamA and the lipoproteins are expressed at a few thousand copies per cell (1.5–6.0×10^3^ copies/cell).^[48]^ In our case, overexpression increased the copy number by ≈100‐fold (≈3.8×10^5^ BamA/cell, Figure S11). Therefore, the effect of endogenous lipoproteins on the observed conformational space would be minimal, if any. This expression level is comparable with the results we previously obtained for the cobalamin transporter BtuB^[19]^ as well as the native expression of some of the outer membrane proteins (OmpA 1×10^5^, OmpC, and OmpF 2×10^4^ copies/cell).[^48^, ^49^] The OM is a protein rich bilayer and normally the OMPs occupy up to 50 % of the surface area.^[50]^ Considering the enormous amount of proteins present in the OM, the expression level for BamA achieved here would not significantly affect the membrane properties. The largest opening of the lateral gate (LO^SB^) was observed while BamA was cross‐linked with the substrate (another BamA, but without the POTRA domains).^[8]^ Although we observed this opening in the native membranes (L1‐β16, Figure 3 C), our extensive measurements on several singly labeled variants gave no evidence for the presence of any significant fraction of such dimers (Figure S6A‐B). Therefore, the dimer might be short‐lived without cross‐linking or the lipoproteins are required for the dimerization.

Considering the low endogenous levels of the Bam proteins, majority of the overexpressed BamA might not be bound with other substrates. Therefore, we suggest that the observed conformational heterogeneity reflects an intrinsically dynamic behavior of BamA in the native membranes rather than a collection of specific snapshots while being “caught in the act”. Supporting this notion, data in the LDAO micelles also indicate such a behavior, although at a reduced level (Figure 2; Figures S7A–G and S8A–E). PELDOR experiment requires frozen sample. Freezing (≈1 s) can induce transition of the membrane into the gel phase, which may slowdown the funneling of the population into the low energy state(s) (if there exist significant energy barriers between different conformations) or in effect would freeze the heterogeneity observed close to the physiological temperature.

In the lateral‐open state, the POTRA5 is observed in a closed conformation with respect to the BamA barrel (Figure 1 B,C). We did not observe this conformation despite the presence of a significant population of BamA with an open lateral gate in the native OM (Figure 3 C, E). Thus, the lipoprotein(s) might be required for coupling the conformational changes of the barrel with the POTRA domains and vice versa. It has been shown that BamA alone can fold OMPs in vitro and the lipoproteins may increase the activity to biologically relevant time scales.^[51]^ The lipoproteins together with the POTRA domains may help to populate specific conformation(s) from the dynamic ensemble we observed here for substrate folding and insertion. BamA may accelerate the insertion of pre‐folded OMPs. Alternatively, the folding can occur at the lateral gate,[^8^, ^52^] POTRA domains, or the barrel lumen.[^53^, ^54^] Together with the hydrophobic mismatch near the lateral gate, the broad conformational space we revealed here might lead to a significant perturbation and disorder of the bilayer^[45]^ to allow the release or insertion of folded substrates. The antibiotic darobactin was shown to inhibit BamA by locking it into the IO conformation.^[55]^ Thus, the conformational heterogeneity as we revealed here might be crucial for the key functions of BamA in the cell.^[56]^

## Conclusion

Structures of BamA revealed the barrel in the inward‐open state.[^45^, ^46^, ^47^] However, our results show that in the native membrane BamA alone can occupy different conformational states. It is reassuring to confirm the lateral gate, L3‐L8, T1‐T6, and the T7‐POTRA5 distances corresponding to the different conformations of BamA (which were solved in different detergent micelles) in the native membrane. At the same time, it is also shown that the native environment can populate an equilibrium between those states and may further modulate the structure. Complementary to the developments in the in situ solid‐state NMR spectroscopy,[^57^, ^58^] our approach offers a great opportunity for further investigations of BamA and the BAM complex in the native outer membrane environment.

## Conflict of interest

The authors declare no conflict of interest.

## Supporting information

As a service to our authors and readers, this journal provides supporting information supplied by the authors. Such materials are peer reviewed and may be re‐organized for online delivery, but are not copy‐edited or typeset. Technical support issues arising from supporting information (other than missing files) should be addressed to the authors.

Supporting Information
