# Supplementary material for: Conformational Flexibility of the Protein Insertase BamA in the Native Asymmetric Bilayer Elucidated by ESR Spectroscopy
Source: Angew Chem Int Ed Engl. 2021 Dec 2;61(2):e202113448. doi: 10.1002/anie.202113448 (PMC9299766; doi:10.1002/anie.202113448)
Supplement: Supplementary file 1 — Supporting Information [file ANIE-61-0-s001.pdf]

Angewandte Chemie
Eine Zeitschrift der Gesellschaft Deutscher Chemiker

Supporting Information

Conformational Flexibility of the Protein Insertase BamA in the Native Asymmetric Bilayer Elucidated by ESR Spectroscopy

*Aathira Gopinath and Benesh Joseph**

anie_202113448_sm_miscellaneous_information.pdf

Table of Contents

Experimental Procedures ... 3
Plasmid construction and mutagenesis ... 3
BamA expression ... 3
Purification and spin labeling of BamA ... 3
Outer membrane isolation and spin labeling ... 3
Spin labeling in *E. coli* ... 3
SDS PAGE and Western Blot ... 3
In vivo complementation growth assay ... 4
CW ESR spectroscopy ... 4
Pulsed ESR spectroscopy ... 4
Figure S1. Purification of BamA into detergent micelles.. ... 5
Figure S2. Room temperature cw ESR spectroscopy of spin labeled BamA variants. ... 6
Figure S3. Characterization of BamA variants in the native outer membrane. ... 7
Figure S4. Colony growth assay for BamA variants in *E. coli* JCM166 cells. ... 8
Figure S5. The transversal relaxation curves for spin labeled BamA variants ... 9
Figure S6. PELDOR measurement of MTSL labeled single cysteine variants in the native OM.. ... 10
Figure S7. PELDOR data analysis for detergent solubilized BamA samples. ... 11
Figure S8. PELDOR spectroscopy of biological replicates for the detergent solubilized BamA. ... 12
Figure S9. PELDOR data analysis of native outer membrane BamA samples. ... 13
Figure S10. PELDOR spectroscopy of biological replicates for the native outer membrane samples. ... 14
Figure S11. Qunatification of BamA in *E. coli* cells. ... 15
Supporting Table 1. Error estimation for the analysis of PELDOR data.. ... 16
References ... 17
Author Contributions ... 17

Experimental Procedures

Plasmid construction and mutagenesis

The *bamA* gene containing a N-terminal His_6 tag and a thrombin cleavage site, both after the signal sequence was custom synthesized (GeneArt, Thermo Fisher Scientific) and subsequently cloned into the pCDFDuet-1 vector. The native cysteines (C690 and C700) were mutated to serines, which was shown to have no negative effect on the function.[1] Cysteines were introduced at the desired positions using the Q5 Site-directed mutagenesis kit (New England Biolabs).

BamA expression

The plasmid containing *bamA* gene was transformed into *E. coli* Rosetta2(DE3) cells and grown in LB media containing 50 µg/ml streptomycin and 34 µg/ml chloramphenicol until an OD_{600} of 0.6-0.8. The culture was then diluted 1:100 times into an autoinduction medium and grown at 37 °C to an OD_{600} of approximately 1.0. Cells were further grown at 18 °C for 12-14 h.

Purification and spin labeling of BamA

Cell culture was spun down at 8000xg for 10 min, resuspended in lysis buffer (50 mM Tris-HCl pH 8.0, 2 mM EDTA, 100 µg/ml lysozyme, 1 mM PMSF, and 1 µg/ml DNaseI) and lysed by sonication. The lysate was pelleted down at 10,000xg for 20 minutes to remove the cell debris. The supernatant was then ultracentrifuged at 200,000xg for 1 hour to collect the membrane fraction. The resulting membranes were solubilized in a buffer containing 50 mM Tris-HCl pH 8.0, 150 mM NaCl, 10 mM imidazole, 2% LDAO and stirred at 4°C for 1 hour. The solution was then centrifuged at 10,000xg for 1 hour to remove aggregates. The supernatant was incubated with Ni Sepharose High Performance slurry (GE Healthcare) for 1 hour at 4°C and subsequently loaded onto a PD-10 Empty column (GE Healthcare). Column was washed with 20 column volume of buffer A (50 mM Tris-HCl pH 8.0, 150 mM NaCl, 25 mM imidazole, and 0.1 % LDAO) and eluted with buffer B (50 mM Tris-HCl pH 8.0, 150 mM NaCl, 300 mM imidazole, and 0.1 % LDAO) The eluted protein was then buffer exchanged into 50 mM Tris-HCl pH 8.0, 150 mM NaCl, and 0.1 % LDAO using PD-10 desalting column (GE Healthcare). Labeling was carried out at this point using a 40-fold excess of 1-oxyl2,2,5,5-tetramethyl-3-pyrroline-3-methyl methanethiosulfonate (MTSL, Toronto Research Chemicals) at room temperature for 30 minutes. The protein was concentrated (Vivaspin 6, MWCO 50,000 Da) and applied to a Superdex 200 Increase10/300 GL column (GE Healthcare). The peak fractions were collected and concentrated to 20 – 40 µM protein.

Outer membrane isolation and spin labeling

BamA native membrane samples were prepared as described earlier.[2] Briefly, the total membrane (approx. 0.5 g) was resuspended into a buffer containing 50 mM Tris-HCl pH 8.0 and 150 mM NaCl. The inner membrane fraction was solubilized by adding 0.5 % N-Lauroylsarcosine sodium salt. The solution was then ultracentrifuged at 200,000xg for 1.5 h. The outer membrane was resuspended in 10 ml of the same buffer. Spin labeling was performed with 10 µM MTSL at room temperature for 1 h. Excess MTSL was then washed off by several rounds of ultracentrifugation and resuspension. The final pellet was resuspended in 50 – 100 µl of the buffer.

Spin labeling in *E. coli*

BamA was expressed in Rosetta2 (DE3) as described earlier.[3] Cells were collected and resuspended in 1 ml buffer containing 45 mM MOPS pH 7.5 and 55 mM NaCl to an OD_{600} of 5.0. Cells were labelled by incubating with 100 µM MTSL for 15 minutes at 25 °C. Cells were then pelleted and resuspended in the same buffer (2x) to remove free MTSL. For the CW ESR experiment, cells were resuspended in 25 µl of the buffer to a final OD_{600} of 200.

SDS PAGE and Western Blot

The samples were prepared by mixing with 5x SDS loading dye (250 mM Tris HCl pH 8.0, 10% SDS, 30% glycerol, 10 mM DTT, and 0.05% bromophenol blue) and boiling at 99°C for 10 min. Afterwards, samples were run on a 4-12% polyacrylamide gel using TEA-Tricine SDS Running Buffer (TruPAGE™, Sigma Aldrich) at 180 V. For western blotting, protein was transferred to PVDF membrane (Amersham, GE Healthcare) by running at 20 V in 1x transfer buffer (12.5 mM Trizma base, 96 mM glycine, and 10% methanol).

Staining was performed using WesternBreeze Chromogenic immunodetection kit (Invitrogen). SDS-PAGE and western blots were imaged using the Gel Doc™ EZ Imager (Bio-Rad).

In vivo complementation growth assay

The activity of BamA mutants was tested using in vivo complementation in JCM166 cells (provided by Sebastian Hiller).[4, 5] The plasmids were transformed into JCM166 cells and plated to LB agar containing 50 µg/ml spectinomycin and 0.05% arabinose. A single colony was picked from each mutant and grown in LB media containing 50 µg/ml spectinomycin and 0.05% arabinose at 37°C till an OD_{600} of approximately 1.5. Culture was pelleted down at 4000xg for 10 min and washed with LB media twice to remove the free arabinose. The final pellet was made into an OD_{600} value of 1.0 and serially diluted into 1:10, 1:100, 1:1000, 1:10000, and 1:100000 folds. A 2 µl of each dilution was plated into LB agar plates with or without 0.05% arabinose.

CW ESR spectroscopy

Continuous-wave ESR measurements were performed at room temperature using a Bruker EMXnano benchtop spectrometer operating at X-band frequency. Measurements were done using a 25 – 40 µL sample in 0.86- or 1.2-mm diameter micropipettes (BRAND, Germany) with 100 kHz modulation frequency, 0.6-2 mW microwave power, 0.15 mT modulation amplitude, and 18 mT sweep width.

Pulsed ESR spectroscopy and data analysis

All the experiments were performed on a a Bruker Elexsys E580 Q-Band Pulsed ESR spectrometer with SpinJet AWG, which was recently installed in our work group. It is equipped with an arbitrary waveform generator (AWG), a 50 W solid state amplifier, a continuous-flow helium cryostat, and a temperature control system (Oxford Instruments). A 15-20 µL sample (20 – 40 µM protein) containing 20% d_8-glycerol was transferred into 1.6 mm outer diameter quartz EPR tubes (Suprasil, Wilmad-LabGlass) and snap-frozen in liquid nitrogen. Phase memory time (T_M) was determined using a 48 ns $\pi/2$–τ– π Gaussian pulse sequence at 50 K with a two-step phase cycling, while τ was increased in 4 ns steps. Instantaneous diffusion was probed in a similar manner while gradually changing the flip angle of the π pulse from π to $\pi/6$. PELDOR measurements were performed with a Bruker EN5107D2 dielectric resonator at 50 K using a dead-time free four-pulse sequence and a 16-step phase cycling ($x[x][x_p]x$).[6, 7] A 38 ns Gaussian pulse (FWHM of 16.1 ns) was used as the pump pulse with a 48 ns (FWHM of 20.4 ns) Gaussian observer pulses. The observer pulses were set at 80 MHz lower than the pump pulse, which was set to the maximum of the echo-detected field swept spectrum. The deuterium modulations were averaged by increasing the first interpulse delay by 16 ns for 8 steps. The data analysis was performed using Tikhonov regularization (TR) as implemented in the MATLAB-based DeerAnalysis2018 package.[8] For TR, the background function arising from intermolecular interactions were removed from the primary data V(t)/V(0) and the resulting form factors F(t)/F(0) were fitted with a model-free approach to distance distributions. Error estimation for the probability distribution was performed by determining the distances for different background functions through gradually changing the time window and or the dimensionality for the spin distribution (see Supplementary Table 1). Simulation of the time-domain data (form factors) were performed using the MATLAB routines as implemented in the DeerAnalysis2018 software. In silico spin labeling and simulations on the inward-open (PDB 5D0O), lateral open (PDB 5LJO), and lateral-open substrate bound (PDB 6V05) structures were performed using a rotamer library approach using the MATLAB-based MMM2018 software package.[9]

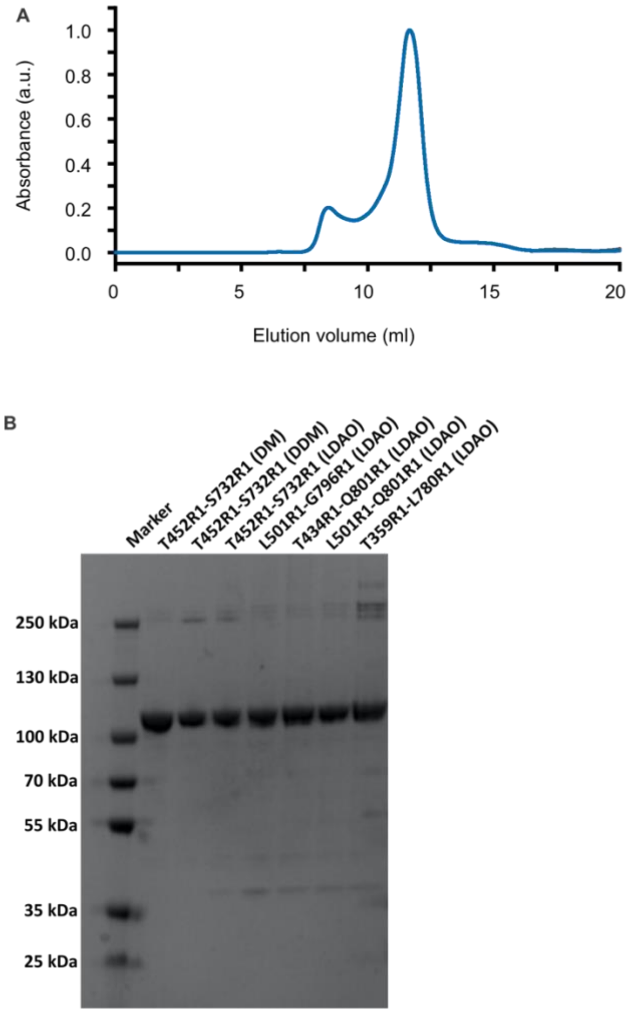

Figure S1. Purification of BamA into detergent micelles. (A) Representative SEC profile of MTSL labeled T434R1-Q801R1 variant in LDAO micelles. PELDOR samples were collected from the major peak fraction and concentrated (when required) to 20 – 40 µM protein. (B) SDS-PAGE analysis of the major peak fractions from SEC showing the bands corresponding to BamA (above the 100 kDa marker) in DM, DDM, or LDAO micelles as indicated.

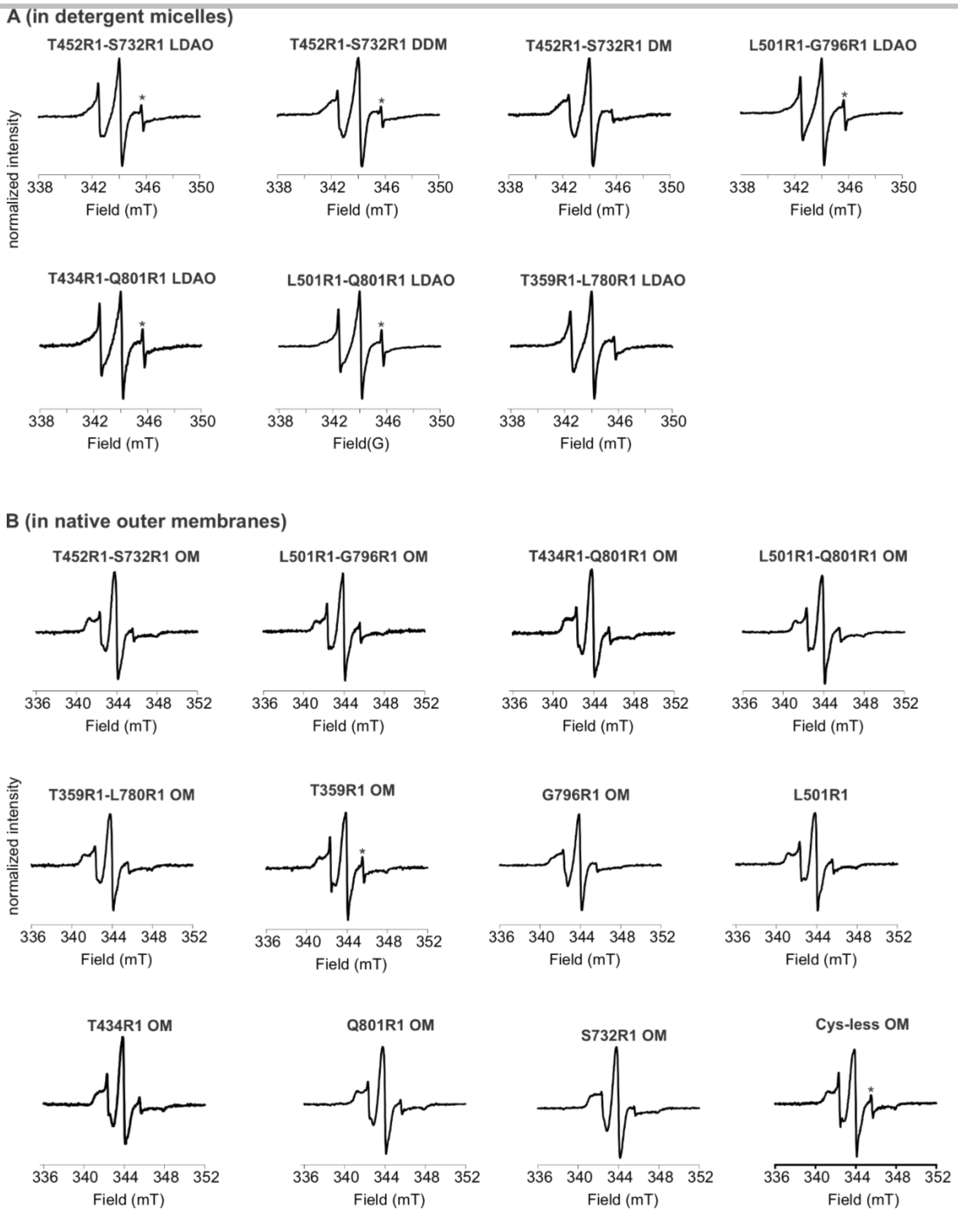

Figure S2. Room temperature cw ESR spectroscopy of spin labeled BamA variants (forming the side chain denoted as R1) in detergent micelles (A) or native outer membranes (B). The tiny amount of free MTSL labels in the final sample when present (which has no effect on the determined distance distribution) is indicated with an asterisk for clarity.

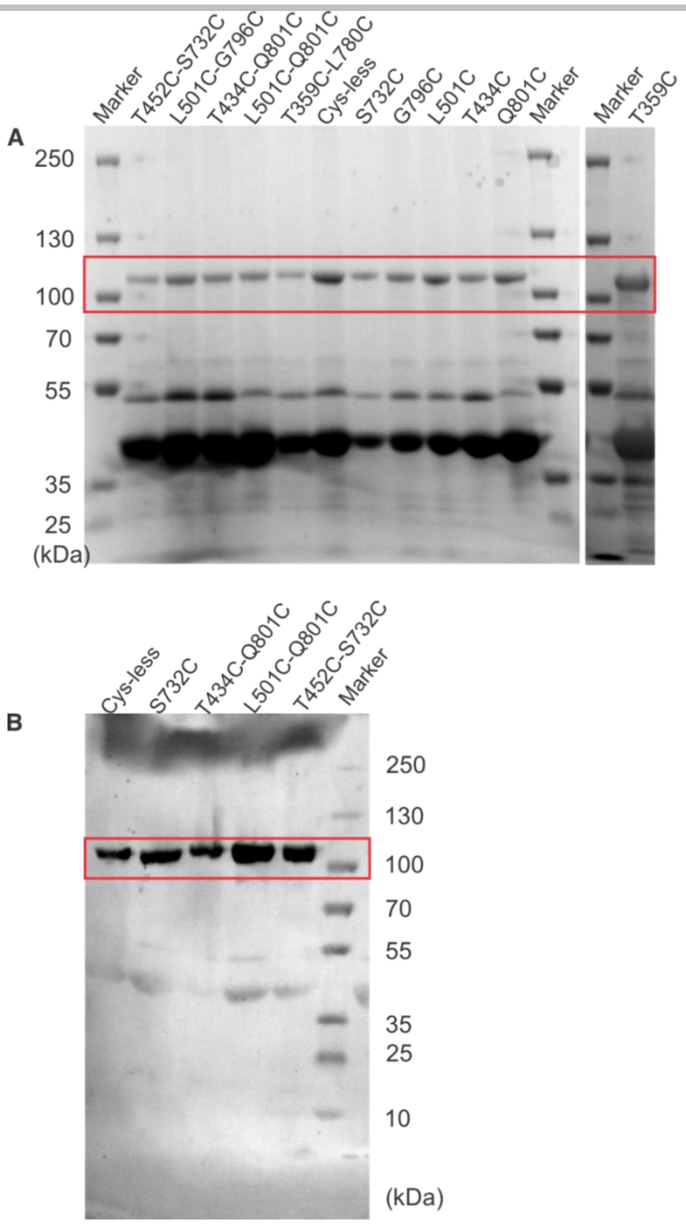

Figure S3. Characterization of BamA variants in the native outer membrane. (A) SDS-PAGE of the purified outer membranes showing the BamA band (highlighted inside the red box) for the single/double cysteine and the Cys-less variants. (B) The BamA band in the outer membranes was further observed using Western blot analysis of the selected variants as indicated.

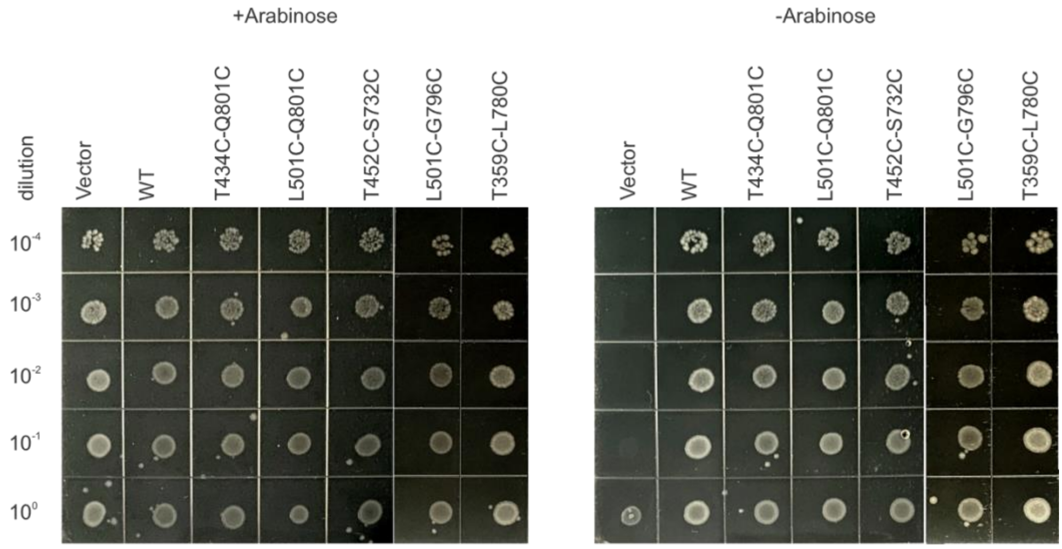

Figure S4. Colony growth assay for BamA variants in *E. coli* JCM166 cells. The WT BamA is gnomically expressed under the control of an arabinose promoter. The cysteine variants (expressed from the corresponding plasmids) gave a similar growth in the absence of arabinose, revealing that the introduced substitutions have no negative impact on BamA function.

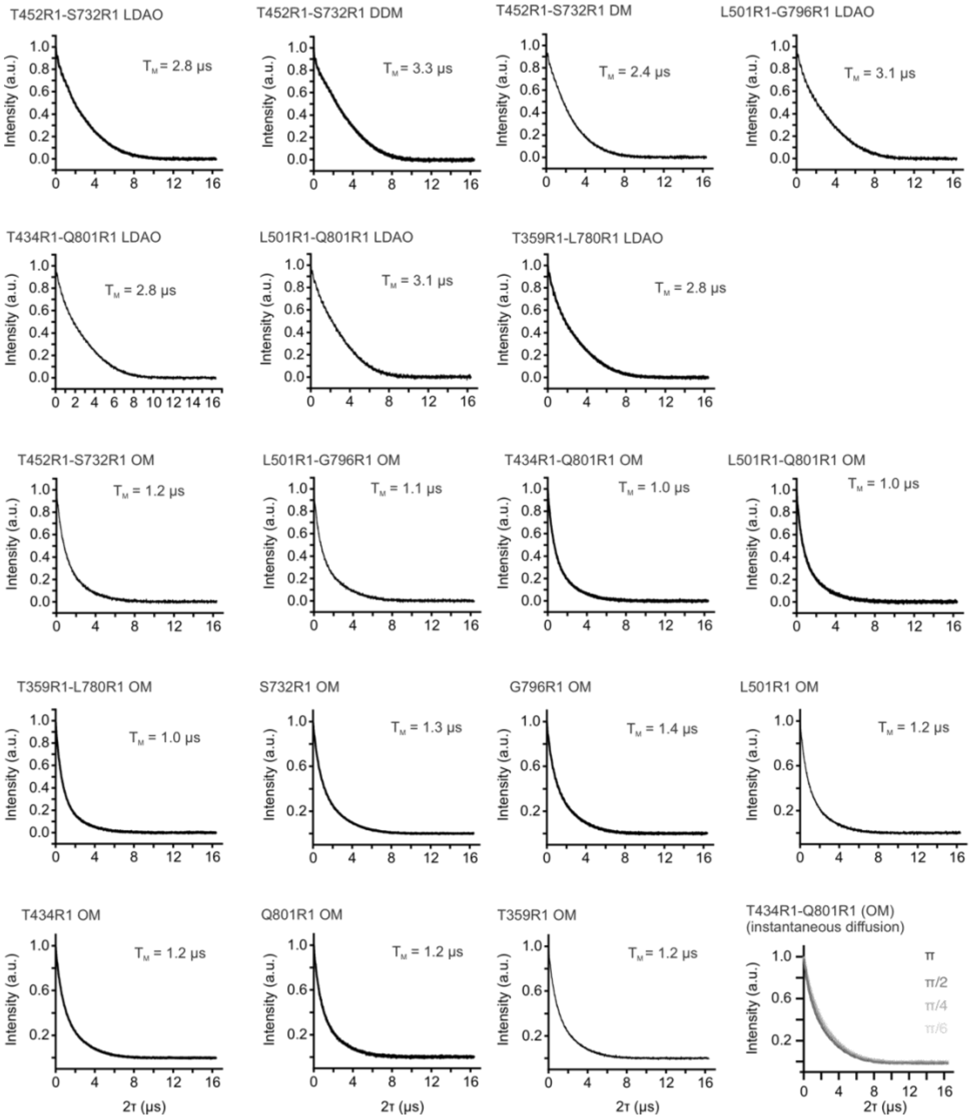

Figure S5. The transversal relaxation curves for spin labeled BamA variants in detergent (DM, DDM, or LDAO as indicated) micelles and the native outer membranes (OM). The last plot at the bottom right shows the relaxation curves (for the T434R1-Q801R1 variant) in the native OM obtained with a π/2–τ– π pulse sequence while gradually changing the flip angle of the π pulse from π to π/6, which altogether ruled out any significant instantaneous diffusion.

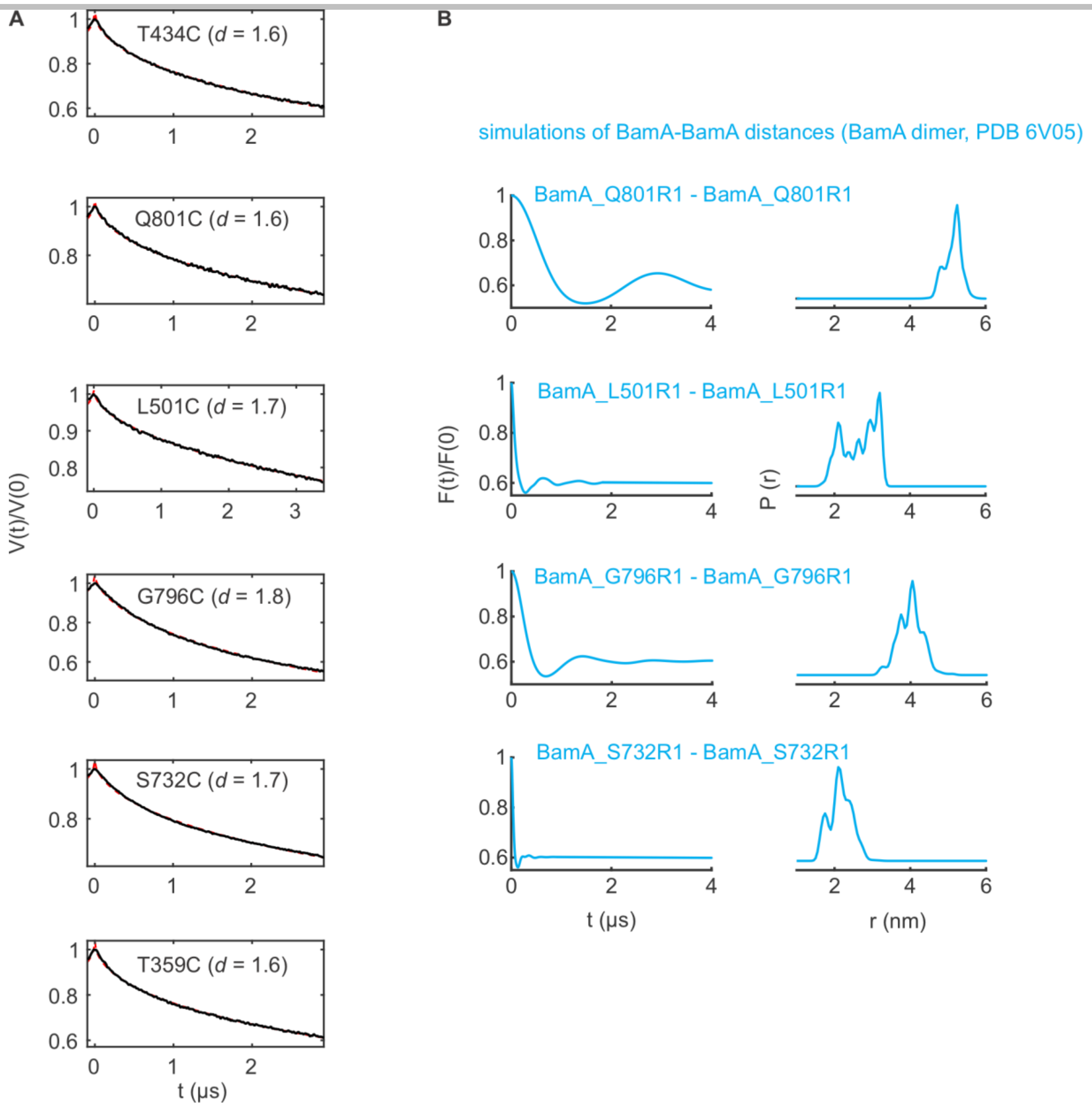

Figure S6. PELDOR measurement of MTSL labeled single cysteine variants in the native OM. (A) The PELDOR data (in black) and the fit (in red) revealed a stretched exponential decay devoid of any distances with a dimensionality for spin distribution, d = 1.6 – 1.8. For membrane samples, a 2D spatial distribution of the spin labels (d = 2.0) is expected in the ideal case (no free spins and homogenous distribution over the membrane). However, the spin labels (on BamA) are not homogenously distributed over the membrane. It has been shown that BamA together with other outer membrane proteins organize into small islands within the membrane.[10] Such an inhomogeneous distribution and spatial organization might account for the somewhat lower value of the d observed. For the double cysteine variants, the data was analyzed using similar background functions and the uncertainty band was estimated by varying the d (between 1.6 – 1.8) and the time window (see Supplementary Table 1). (B) Simulations of the BamA-BamA distances P(r) and the corresponding form factors F(t)/F(0) using the dimer structure, which was stabilized via cysteine cross-linking (PDB 6V05). Position 434 is not resolved and position 359 is absent in one of the monomers, hence the distances could not be simulated. Simulations show distances in the observable range (as for the double cysteine variants), whereas their total absence in the experimental data rule out the existence of any dimers in the native outer membranes. The dimer might be short-lived without cross-linking or the lipoprotein(s) are required for the dimerization.

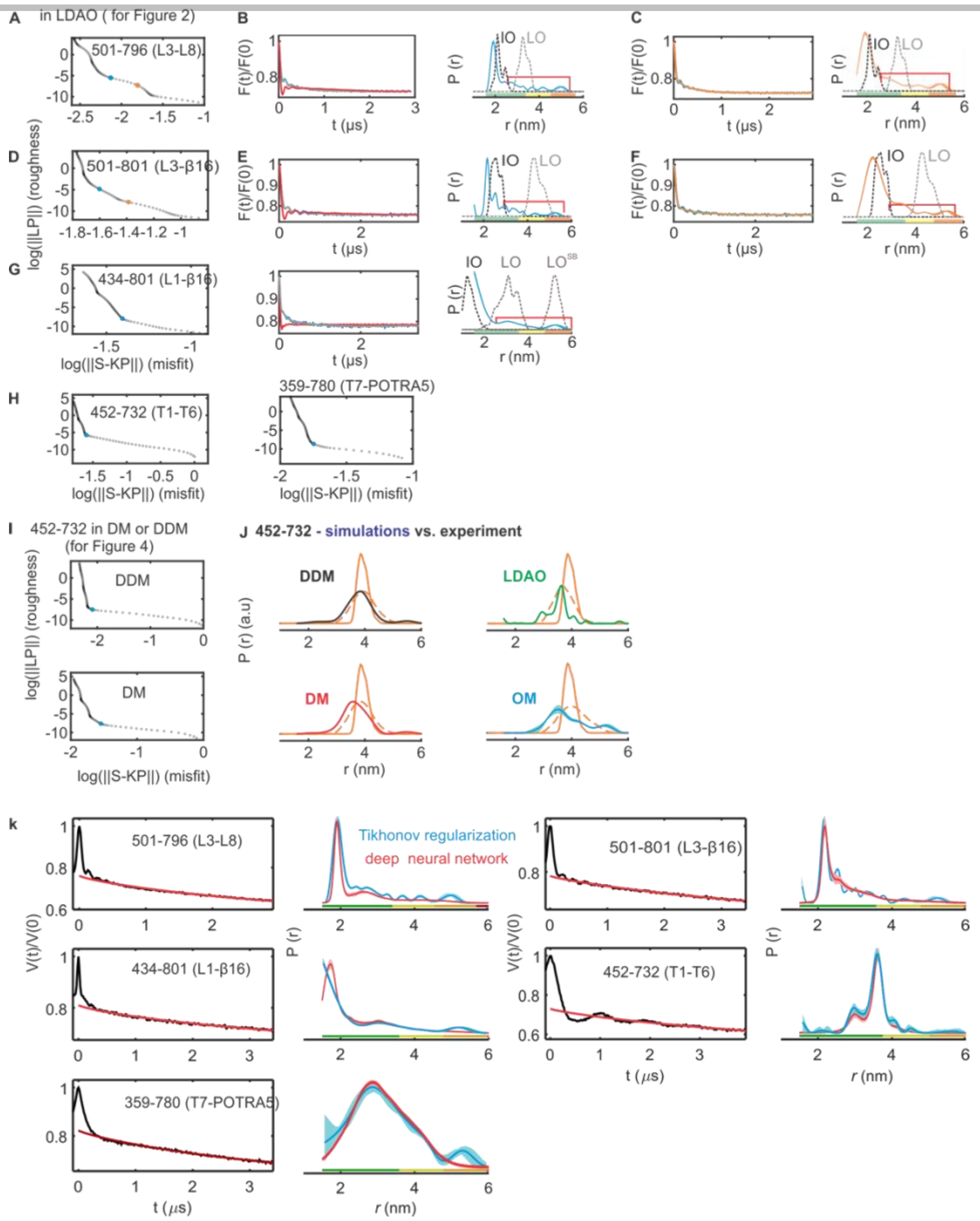

Figure S7. PELDOR data analysis for detergent solubilized BamA samples. The primary data are shown in Figure 2 and panel K below. (A) Left panels, L-curves from Tikhonov regularization for BamA L501R1-G796R1 variant in LDAO micelles with the used regularization points highlighted (see Supplementary Table 1). (B) The form factor (in blue) with the fit (grey) and the distance distribution (right, in blue). The error bars on P(r) show the full variation of the probability for the given distances corresponding to the uncertainty in the background function (see Supplementary Table 1), which is invisible if smaller than the linewidth. Simulations on the inward-open (IO), lateral-open (LO), or the lateral-open substrate bound (LO^{SB}) states are overlaid (in dotted lines). An additional simulation of the form factor after excluding the distances indicated with the red line is overlaid. The observed difference shows that the corresponding distances are resolved in the experimental time-domain data. Nevertheless, due to the small probability amplitudes (especially in the orange zone), there is an uncertainty for the exact distances and the small peaks are only qualitatively interpretable. (C) The form factor was fitted using a higher regularization parameter (α, corresponding to the orange point in panel A). This corresponds to the smallest value of α, which smoothened the fast oscillations in the beginning (which defines the width of the major and the narrow distance peak) and show that those distances (indicated with the red line in panel B) correspond to a rather broad distribution (also see the neural network analysis in panel K). (D, E, and F) Data for the L501R1-Q801R1 variant as described above for panels A, B, and C respectively. (G) Analysis for the T434R1-Q801R1 variant as explained for panels A and B respectively. (H) L-curves for the T452R1-S732R1 or the T359R1-L780R1 variant as indicated. The primary data for the positions above are presented in Figure 2. (I) L-curves for the T452R1-S732R1 variants in DM or DDM as indicated (primary data and form factor is shown in Figure 4). (J) Comparison between experimental distance distributions for T1-T6 (T452R1-S732R1) in different detergent micelles and the simulation (in solid orange line) on the IO structure (PDB 5D0O). The observed discrepancy could be accommodated in a simulation after ignoring the side chain packing around the spin label (in dotted orange line). The experimental distribution in the outer membranes (OM) as well could be matched, although not perfectly, suggesting only a minor alteration in the conformation of the turns, if any (OM distance distribution corresponds to the data presented in Figure S10D). Simulations were performed using the MATLAB-based MMM software.[9] (K) Data analysis (as indicated) using deep neural network processing. For a direct comparison, output P(r) from TR is overlaid. The color code for the probability distributions (P(r)) relates the reliability for different features with the length of the observed dipolar evolution time. In the green zone, shape, width, and the mean distance are accurate. In the yellow zone, width and the mean, and in the orange zone the mean distance are reliable.

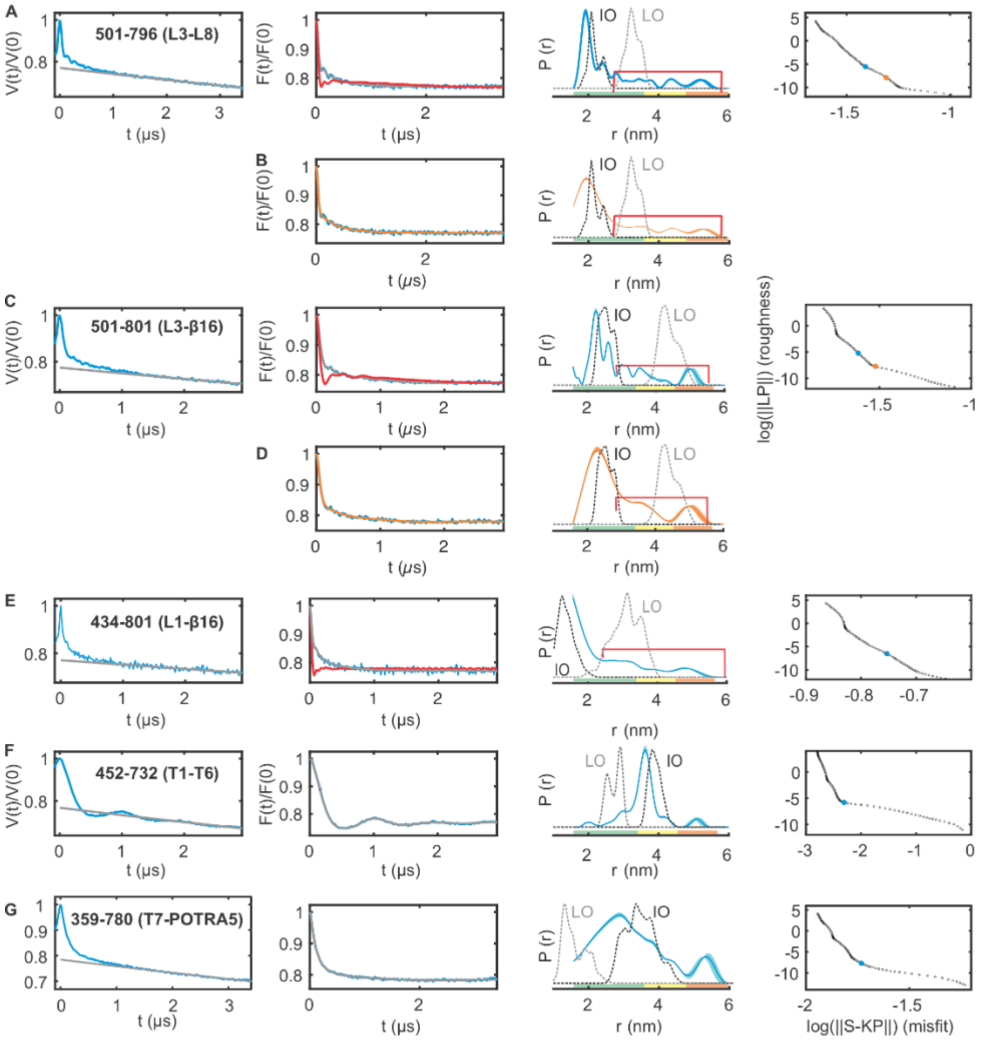

Figure S8. PELDOR spectroscopy of a fully independent set of biological replicates for the detergent (LDAO) solubilized BamA samples. These samples gave results identical to the other data set presented in Figure 2 and Figure S7. (A) The primary PELDOR data (V(t)/V(0), in blue) overlaid with the intermolecular (or background) contribution (in grey), form factors F(t)/F(0) (in blue) with the fits (in grey), probability distribution (P(r)), and the L-curves (the rightmost panels) are shown. The error bars on P(r) show the full variation of the probability for the given distances corresponding to the uncertainty in the background function (see Supplementary Table 1), which is invisible if smaller than the linewidth. A simulation (in red) of the form factor F(t)/F(0) after excluding the distances highlighted with the red lines is given, which shows that those distances are resolved in the time-domain data. Nevertheless, due to the small probability amplitudes (especially in the orange zone), there is an uncertainty for the exact distances and the small peaks are only qualitatively interpretable. (B) The form factor was further fitted (in orange) using a higher regularization parameter (α, corresponding to the orange points in the rightmost panels). This matches to the smallest value of α, which smoothened the fast oscillations in the beginning (which defines the width of the major and the narrow distance peak) and show that those distances (indicated with the red lines) correspond to a rather broad distribution. (C, D) Data analysis for the indicated variant with the description analogous to that for panels A and B. (E, F, and G). Data analysis for the indicated variants with the description analogous to that for panels A except that the additional simulation of F(t)/F(0) is not shown for the last two variants. The color code for the probability distributions (P(r)) corresponds to the description in Figure S7.

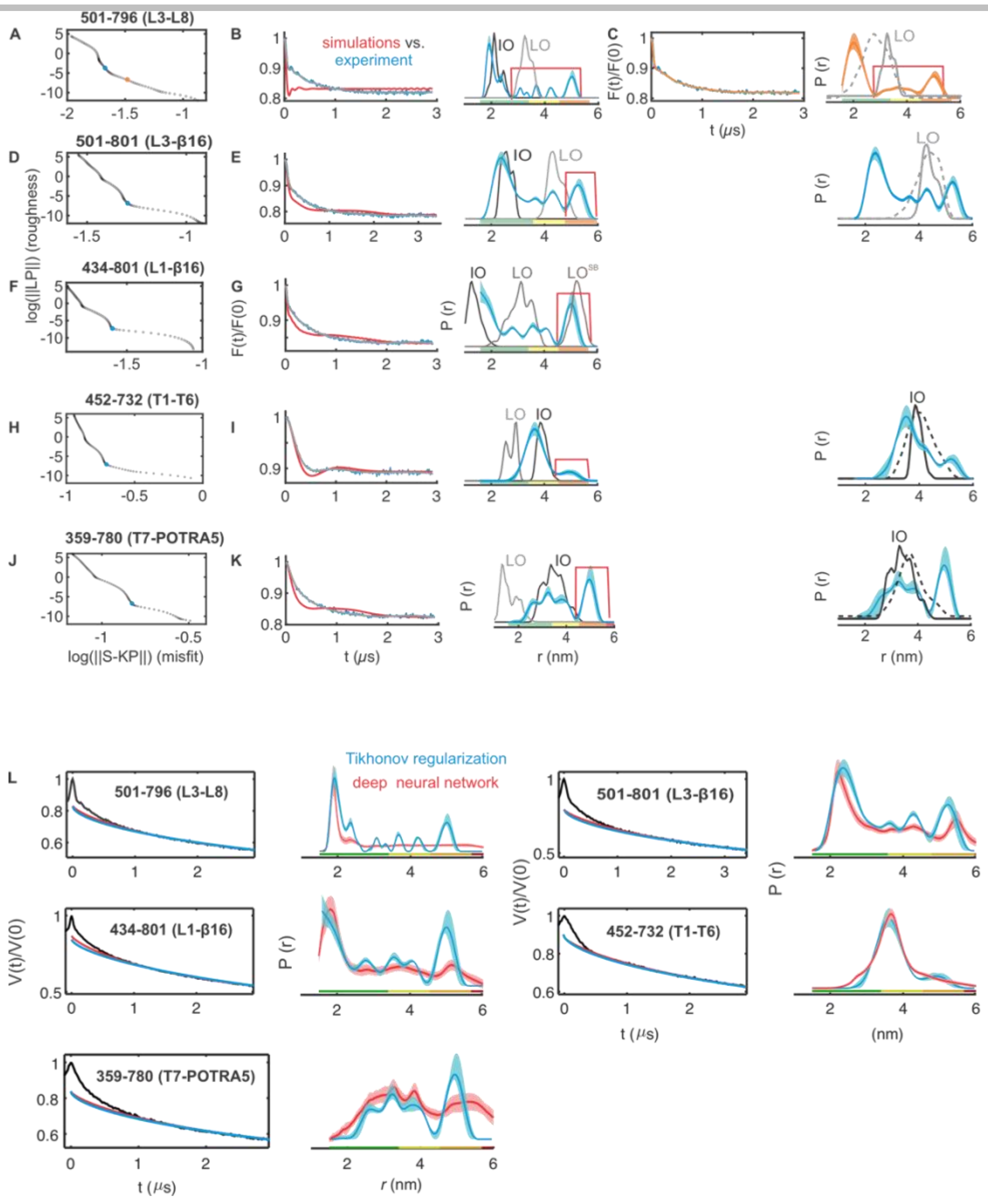

Figure S9. PELDOR data analysis of native outer membrane (OM) BamA samples. (A) Left panels, L-curves from Tikhonov regularization for the indicated variants in LDAO micelles with the used regularization points highlighted (see Supplementary Table 1). The primary data are shown in Figure 3 and panel L below. (B) The form factor (in blue) with the fit (grey) and the distance distribution (right, in blue). The error bars on P(r) show the full variation of the probability for the given distances corresponding to the uncertainty in the background function (see Supplementary Table 1), which is invisible if smaller than the linewidth. Simulations on the inward-open (IO), lateral-open (LO), or the lateral-open substrate bound (LO^{SB}) states are overlaid (in dotted lines). An additional simulation of the form factor after excluding the distances indicated with the red line is overlaid. The observed difference shows that the corresponding distances are resolved in the experimental time-domain data. (C) The form factor was fitted using a higher regularization parameter (α, corresponding to the orange point in panel A). This corresponds to the smallest value of α, which smoothened the fast oscillations in the beginning (which defines the width of the major and the narrow distance peak) and show that those distances (indicated with the red line in panel B) correspond to a rather broad distribution. (D-E, F-G, H-I, and J-K) Data analysis for the indicated variants with the description analogous to that for panels A-B. The distances in the orange zone are further confirmed with a comparison of the simulation of the form factor (in red) after excluding those distances with the experimental data. A comparison of the experimental distance distribution with the simulation (excluding for the T434R1-Q801R1 variant as the overall distribution shows good agreement with the simulations) on the IO structure (in black) or the LO structure (in grey) is shown (rightmost panels). Another simulation after ignoring the side chain packing (to observe the best possible match with experimental distribution) around spin labels is also shown (in dotted black or grey lines). Even the latter simulation cannot fully match the L3-L8, L3-β16, and the T7-POTRA5 experimental distribution, suggesting additional flexibility of these structural elements. (L) Data analysis (as indicated) using deep neural network processing. For the primary data V(t)/V(0), the experimentally determined background functions (in blue, see Figure S6) is overlaid with the neural network predicted background (in red), which overall revealed excellent agreement. For a direct comparison, output P(r) from TR is overlaid. The color code relates the reliability for different features of the probability distribution as explained in Figure S7.

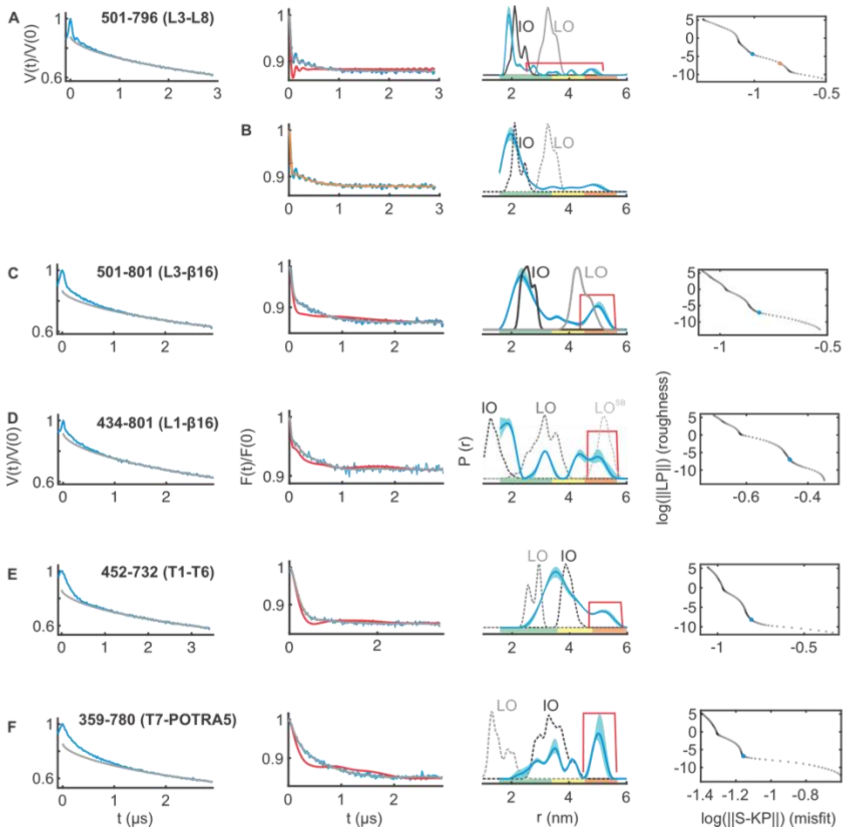

Figure S10. PELDOR spectroscopy of a fully independent set of biological replicates for the native outer membrane samples. These samples gave results similar to the other data set presented in Figure 3. (A) The primary PELDOR data (V(t)/V(0), in blue) overlaid with the intermolecular (or background) contribution (in grey), form factors F(t)/F(0) (in blue) with the fits (in grey), probability distribution (P(r)), and the L-curves (the rightmost panels) are shown. The error bars on P(r) show the full variation of the probability for the given distances corresponding to the uncertainty in the background function (see Supplementary Table 1), which is invisible if smaller than the linewidth. A simulation (in red) of the form factor F(t)/F(0) after excluding the distances highlighted with the red line is given, which shows that those distances are resolved in the time-domain data. (B) The form factor was further fitted (in orange) using a higher regularization parameter (α, corresponding to the orange point in the rightmost panel). This matches to the smallest value of α, which smoothened the fast oscillations in the beginning (which defines the width of the major and the narrow distance peak) and show that those distances (indicated with the red lines) may correspond to a rather broad distribution. The probability amplitude in this range (for this pair) is somewhat lower when compared with the other replicate (Figure 3A and S9A), which might reflect the variability/flexibility of the conformational dynamics within the native environment. (C, D, E, and F) Data analysis for the indicated variants with the description analogous to that for panel A. The distances in the orange zone are further confirmed with a comparison of the simulation of the form factor (in red, after excluding those distances) with the experimental data. The color code for the probability distributions (P(r)) corresponds to the description in Figure S7.

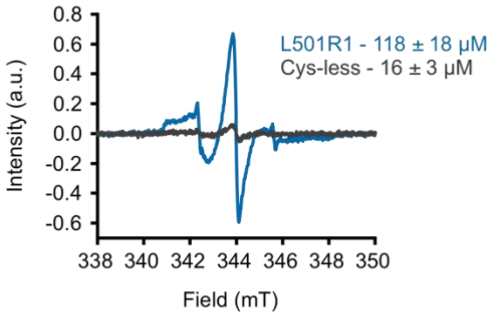

Figure S11. Quantification of BamA expression in *E. coli* cells. The Cys-less and L501C variants were labeled with MTSL in live *E. coli* following overexpression. Measurements were performed with 25 µL sample containing 4 x 10^9 cells. After subtracting the background labeling obtained with the WT, the L501C mutant gave ~100 µM spin, which corresponds to 1.5 x 10^{15} spins (BamA) in the sample. Dividing it with the cell number gives an expression level of ~3.8 x 10^5 BamA/cell. This expression level is comparable with the results we previously obtained for the cobalamin transporter BtuB[11] as well as the native expression of some outer membrane proteins (OmpA 1x10^5, OmpC and OmpF 2x10^4 copies/cell)[12, 13]. Under laboratory conditions, *E. coli* expresses about 1.5 – 4.0 x 10^3 copies of BamA/cell.[12] Thus, in our case the overexpression leads to ~100-fold higher expression. This estimation assumes that all the BamA are labeled in *E. coli* (100% labeling) and a lower labeling efficiency would further increase the calculated expression level. The PELDOR data for the L501R1-Q801R1 variant in the native OM gave a modulation depth (Δ) of ~20% (Figure 3B). This corresponds to ~70% labeling efficiency (with the Δ_{max} = 30% under our experimental set up), although it should be even higher as the background labeling reduces the effective modulation depth. Therefore, the estimated value would be close the actual expression level and may increase by a factor of up to 1.5–fold (70 – 100% labeling efficiency).

SUPPORTING INFORMATION

Supporting Table 1. Error estimation for the analysis of PELDOR data in detergent micelles and the native outer membranes. Data analysis was performed using Tikhonov regularization as implemented in the MATLAB-based DeerAnalysis2018 software.[8] The error bounds corresponding to the uncertainty in the background function was determined by calculating the probabilities for all the distances as a function of different background models. For the detergent solubilized samples, the starting value for the background function was varied in the indicated time window in 11 steps at a fixed value of the dimensionality for the spin distribution (*d* = 3, n.a. means not applicable). For the native membrane samples, the uncertainty bands were calculated from a combined variation of the dimensionality (*d* = 1.6 – 1.8 in 3 steps) and the starting value for the background function (in the indicated range in 11 steps). This range of *d* corresponds to the values experimentally determined for several singly labeled variants (see Figure S6). Another independent neural network analysis as well predicted the same background (or *d*, Figure S9L). The data were pruned at a factor (L_{prune}) of 1.15 of the r.m.s.d from the best fit. For each sample, details for two biologically independent replicates and the corresponding figures are indicated. Small deviation for the regularization parameter (α, when present) between the replicates is attributed to the differences in the S/N or the variability in the biological environment.

Sample	Figure(s)	Error estimation/validation					Regularization parameter (α)
		dimensionality (*d*)		starting time window			
		value/range	steps	t_{max} (μs)	range (ns)	steps	
**In detergent micelles**							
L501R1-G796R1 (LDAO)	2, S7	3.0	n.a.	2.9	576 – 1744	11	7.9
L501R1-G796R1 (LDAO)	S8	3.0	n.a.	3.4	699 – 2048	11	12.6
L501R1-Q801R1 (LDAO)	2, S7	3.0	n.a.	3.4	699 – 2049	11	5.0
L501R1-Q801R1 (LDAO)	S8	3.0	n.a.	2.9	592 – 1760	11	5.0
T434R1-Q801R1 (LDAO)	2, S7	3.0	n.a.	3.4	699 – 2048	11	158
T434R1-Q801R1 (LDAO)	S8	3.0	n.a.	2.9	576 – 1744	11	158
T452R1-S732R1 (LDAO)	2, S7	3.0	n.a.	3.9	784 – 2352	11	20
T452R1-S732R1 (LDAO)	S8	3.0	n.a.	2.9	592 – 1760	11	20
T452R1-S732R1 (DM)	4, S7	3.0	n.a.	3.5	688 – 2048	11	158
T452R1-S732R1 (DDM)	4, S7	3.0	n.a.	3.5	688 – 2048	11	126
T359R1-L780R1 (LDAO)	2, S7	3.0	n.a.	3.4	699 – 2048	11	200
T359R1-L780R1 (LDAO)	S8	3.0	n.a.	3.4	688 – 2048	11	100
In the native outer membrane (OM)							
L501R1-G796R1 (OM)	3, S9	1.6 – 1.8	3	2.9	576 – 1744	11	7.94
L501R1-G796R1 (OM)	S10	1.6 – 1.8	3	2.9	576 – 1744	11	6.31
L501R1-Q801R1 (OM)	3, S9	1.6 – 1.8	3	2.9	576 – 1744	11	126
L501R1-Q801R1 (OM)	S10	1.6 – 1.8	3	2.4	490 – 1440	11	126
T434R1-Q801R1 (OM)	3, S9	1.6 – 1.8	3	2.9	576 – 1744	11	63.1
T434R1-Q801R1 (OM)	S10	1.6 – 1.8	3	2.9	578 – 1744	11	50.1
T452R1-S732R1 (OM)	3, S9	1.6 – 1.8	3	2.9	688 – 2048	11	126
T452R1-S732R1 (OM)	S10	1.6 – 1.8	3	3.4	576 – 1744	11	251
T359R1-L780R1 (OM)	3, S9	1.6 – 1.8	3	2.9	592 – 1760	11	50.1
T359R1-L780R1 (OM)	S10	1.6 – 1.8	3	2.9	592 – 1760	11	79.4

References

[1] M. G. Iadanza, A. J. Higgins, B. Schiffrin, A. N. Calabrese, D. J. Brockwell, A. E. Ashcroft, S. E. Radford, N. A. Ranson, *Nat. Commun.* **2016**, *7*.
[2] B. Joseph, E. A. Jaumann, A. Sikora, K. Barth, T. F. Prisner, D. S. Cafiso, *Nat. Protoc.* **2019**, *14*, 2344-2369.
[3] S. Ketter, B. Joseph, A. Gopinath, O. Rogozhnikova, D. Trukhin, V. M. Tormyshev, E. G. Bagryanskaya, *Chem. Eu. J.* **2020,** *27,* 2299-2304
[4] T. Wu, J. Malinverni, N. Ruiz, S. Kim, T. J. Silhavy, D. Kahne, *Cell* **2005**, *121*, 235-245.
[5] L. Xiao, L. Han, B. Li, M. Zhang, H. Zhou, Q. Luo, X. Zhang, Y. Huang, *FASEB J.* **2021**, *35*, e21207.
[6] M. Pannier, S. Veit, A. Godt, G. Jeschke, H. W. Spiess, *J. Magn. Reson.* **2000**, *142*, 331-340.
[7] C. E. Tait, S. Stoll, *Phys. Chem. Chem. Phys.* **2016**, *18*, 18470-18485.
[8] G. Jeschke, V. Chechik, P. Ionita, A. Godt, H. Zimmermann, J. Banham, C. R. Timmel, D. Hilger, H. Jung, *Appl. Magn. Reson. 30*, **2006**, 473-498.
[9] Y. Polyhach, E. Bordignon, G. Jeschke, *Phys. Chem. Chem. Phys.* **2011**, *13*, 2356-2366.
[10] P. Rassam, N. A. Copeland, O. Birkholz, C. Toth, M. Chavent, A. L. Duncan, S. J. Cross, N. G. Housden, R. Kaminska, U. Seger, D. M. Quinn, T. J. Garrod, M. S. Sansom, J. Piehler, C. G. Baumann, C. Kleanthous, *Nature* **2015**, *523*, 333-336.
[11] B. Joseph, A. Sikora, E. Bordignon, G. Jeschke, D. S. Cafiso, T. F. Prisner, *Angew. Chem. Int. Ed.* **2015**, *54*, 6196-6199.
[12] G. W. Li, D. Burkhardt, C. Gross, J. S. Weissman, *Cell* **2014**, *157*, 624-635.
[13] B. Soufi, K. Krug, A. Harst, B. Macek, *Front. Microbiol.* **2015**, *6*, 103.

Author Contributions

A.G. performed all the biochemical experiments. A.G. and B.J. performed the ESR experiments and analyzed the data. A.G. and B.J. wrote the paper. B.J. conceived the idea, acquired the funding, and supervised/administered the project.
